# A multilocus sequence analysis scheme for characterization of *Flavobacterium columnare* isolates

**DOI:** 10.1186/s12866-015-0576-4

**Published:** 2015-10-30

**Authors:** Roghaieh Ashrafi, Katja Pulkkinen, Lotta-Riina Sundberg, Nina Pekkala, Tarmo Ketola

**Affiliations:** Centre of Excellence in Biological Interactions, Department of Biological and Environmental Science, University of Jyvaskyla, P.O. Box 35, Jyväskylän yliopisto, FI-40014 Finland; Department of Biological and Environmental Science, University of Jyvaskyla, P.O. Box 35, Jyväskylän yliopisto, FI-40014 Finland

**Keywords:** Flavobacterium columnare, MLST/MLSA scheme, ClonalFrame, Recombination rate, Clonality

## Abstract

**Background:**

Columnaris disease caused by *Flavobacterium columnare* is a serious problem in aquaculture, annually causing large economic losses around the world. Despite considerable research, the molecular epidemiology of *F. columnare* remains poorly understood.

**Methods:**

We investigated the population structure and spatiotemporal changes in the genetic diversity of *F. columnare* population in Finland by using a multilocus sequence typing (MLST) and analysis (MLSA) based on DNA sequence variation within six housekeeping genes. A total of 83 strains of *F. columnare* were collected from eight different areas located across the country between 2003 and 2012.

**Results:**

Partial sequencing of six housekeeping genes (*trpB*, *tuf*, *atpA*, *rpoD*, *gyrB* and *dnaK*) revealed eight sequence types and a moderate level of genetic diversity (H = 0.460). Phylogenetic analysis of the concatenated protein-encoding gene sequence data (ca. 3,509 nucleotides) formed two lineages, which could be further divided into five clusters. All analysed *F. columnare* strains appeared to have a genetic origin distinct from that of another important fish pathogen form the genus Flavobacterium, *F. psychrophilum*. Although the value of the index of association between alleles, 0.292 (*P* < 0.001), supports some degree of clonality for this species in Finland, recombination has introduced molecular diversity to the population almost three times more than mutation.

**Conclusion:**

The results suggest that Finnish *F. columnare* strains have an epidemic population structure followed by clonal expansion of successful genotypes. Our study with reproducible methodology and comparable results establishes a robust framework for the discrimination and phylogenetic analysis of *F. columnare* isolates, which will help to improve our understanding about geographic distribution and epidemiology of columnaris disease.

**Electronic supplementary material:**

The online version of this article (doi:10.1186/s12866-015-0576-4) contains supplementary material, which is available to authorized users.

## Background

*Flavobacterium columnare* is a Gram-negative bacterium belonging to the family *Flavobacteriaceae* (phylum Bacteroidetes) [1]. Columnaris disease caused by *F. columnare* represents a continuous threat to the growing aquaculture industry worldwide. It has been ranked as the second most common disease of the channel catfish (*Ictalurus punctatus*) industry in the United States [2, 3]. The bacterium is capable of causing infections in both warm and cold water species of fish [4], and it infects fish species around the world, including carp, channel catfish, goldfish, eel, perch, tilapia, pike perch, rainbow trout, brown trout, salmon, tiger muskellunge and walleye [2, 5]. *F. columnare* causes epidermal infections affecting gills, skin and fins of the fish, producing either acute or chronic infections, depending on the virulence and genetic group (genomovar) of the strain, as well as on environmental [6] and host-related factors [2, 5, 7].

*F. columnare* has high phenotypic homogeneity, therefore strain characterization by standard biochemical tests is not appropriate [8, 9]. However, *F. columnare* has been divided into three genomovars (I, II, III) using analysis of 16S rDNA by restriction-fragment length polymorphism (16S rDNA-RFLP) [10]. A recent study further increased the resolution of the method to identify a new genomovar (II/III) [11]. Of these, genomovars I and II have been reported in Europe as either common European (genomovar I) or likely imported (genomovar II or Asian type strains) [12]. To obtain higher resolution on genetic diversity of *F. columnare,* several other molecular typing approaches have been used, including single-strand conformation polymorphism (SSCP) [13], amplified fragment length polymorphism (AFLP) [14], pulsed-field gel electrophoresis (PFGE) [3], automated ribosomal intergenic spacer analysis (ARISA) [8] and internal spacer region-single strand conformation polymorphism analysis (ISR-SSCP) [15]. Although the overall discriminatory power of these methods is high, they can suffer from poor inter-laboratory interpretability, and they are not suitable for population structure studies. Moreover, these genetic markers accumulate genetic variation rapidly, which can interfere with investigating evolutionary phylogenetic relationships or global epidemiology between closely related species of bacteria [9, 16] .

In 2012, the complete genome of *F. columnare* was published [17], making it possible to compare genes from individual isolates for developing multilocus sequence typing (MLST) and multilocus sequence analysis (MLSA) schemes. MLST/MLSA schemes provide portable, universal, highly discriminatory and unambiguous data [18–20]. Because this method indexes variation in housekeeping genes that have a relatively slow evolutionary rate, it has been widely used to infer population genetic structure of several different bacterial groups [19, 21–23].

The MLST method typically uses variation in four to seven housekeeping gene sequences to characterize isolates of bacterial species. The allele-based MLST method relies on allelic profiles (the specific combination of alleles for each isolate) and sequence type designations (isolates with an identical allelic profile) to estimate relatedness among isolates and so it ignores the number of nucleotide differences between alleles. The MLSA method, however, relies directly on nucleotide sequences; it uses concatenated sequences of fragments of housekeeping genes to determine genus-wide phylogenetic relationships. Nevertheless, it has been shown that MLSA can also provide robust resolution at the intraspecific level, especially when inadequate phylogenetic resolution prevents MLST from distinguishing phylogenetically closely related strains [24, 25].

Since the first columnaris outbreak in Finland in 1984, *F. columnare* has been reported as a major threat to salmonid fish farming, particularly rainbow trout (*Oncorhynchus mykiss*) [7]. Despite its importance as a fish pathogen, the genetic diversity and population structure of *F. columnare* are poorly known. The genetic characterization of *F. columnare* is essential not only to develop appropriate management strategies to minimize the risk of columnaris disease in Finnish fish farms but also to better understand host specificity, pathogenicity, and distributional pattern of this bacterium, which is critical for understanding the emergence of columnaris outbreaks worldwide. This prompted us to develop the first MLST/MLSA scheme for this species in order to investigate the population structure of *F. columnare* strains isolated from different geographic areas in Finland.

## Materials and Methods

### Bacterial strains and culture conditions

**Fig. 1:**
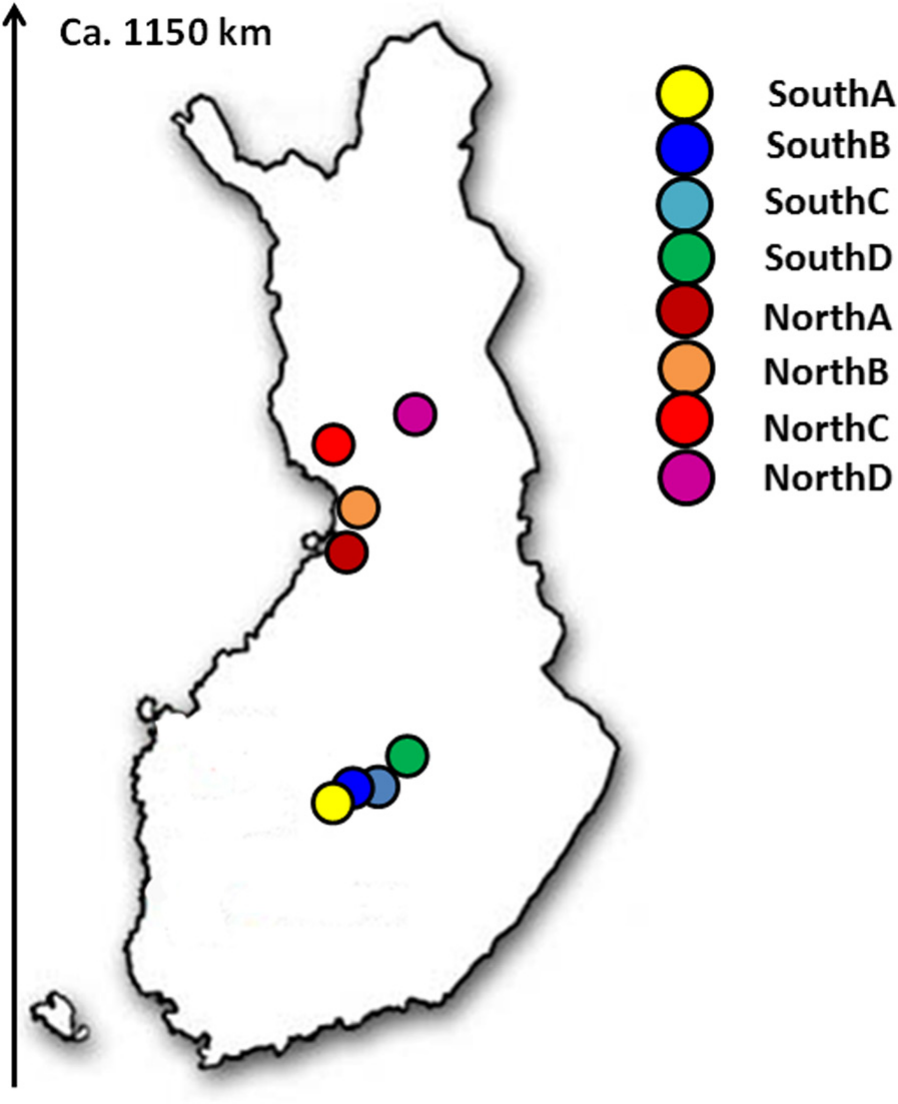
Location of sample sites in Finland for the 83 *F. columnare* isolates used in this study

From 2003 to 2012, 83 *F. columnare* strains were obtained from nine different fish species (*n* = 59) and water samples (*n* = 28), in eight geographic locations in Finland comprising four northern and four southern locations (Additional file 1 and Fig. 1). It is worth noting that all Finnish *F. columnare* isolates have been assigned to genomovar I, [8]. The *F. columnare* type strain NCIMB 2248^T^ isolated in the USA and two reference strains JIP39/87 and ATCC49512, both isolated in France, were also included in the sample collection. The sequences for strain ATCC 49512 has been retrieved from GenBank using their accession numbers. All the Finnish strains were originally isolated using standard culture methods including Shieh medium [26], Shieh medium supplemented with tobramycin [27], or AO-agar [28]. Pure cultures were stored frozen at −80 °C in Shieh medium containing 10 % of glycerol and 10 % of fetal calf serum. Before genomic DNA extraction, the strains were revived from the stocks in 3 ml of Shieh medium for 24 h while shaking (200 rpm) at 25.5 °C. Overnight cultures were diluted into fresh Shieh medium (1:10) and allowed to regrow at 25.5 °C on the shaker for another 24 hours.

### DNA extraction, PCR amplification and sequencing of housekeeping genes

Bacterial cells were harvested from fresh cultures by centrifugation at 6000 x g for 10 min. Bacterial genomic DNA was extracted using the Wizard Genomic DNA Purification Kit (Promega, USA). The candidate genes for this study (*trpB*, *gyrB*, *dnaK*, *fumC*, *murG, rplB*, *recA, tuf*, *atpA*, *glyA*, *rpoD*, 16S rDNA and *fstQ*) were chosen based on the previously published MLST scheme for *Flavobacterium psychrophilum* by Nicolas et al. [22]. We used the corresponding sequences derived from the whole genome sequence of *F. columnare* ATCC 49512 (= CIP 103533 = TG 44/87) [17] to redesign the primers for *F. columnare.* The primers were designed using Primer3 (v.0.4.0) [29, 30] and checked for specificity using the NCBI Primer-BLAST tool (http://www.ncbi.nlm.nih.gov/tools/primer-blast/). All primers used in this study are listed in Additional file 2.

PCR reactions were performed in a total volume of 20 μl containing 1X Phusion Flash PCR Master Mix (Thermo Scientific), 0.5 μM forward primer, 0.5 μM reverse primer, and 100 ng of genomic DNA. PCR reactions were performed on a BioRad 1000C thermal cycler, under the following conditions: 98 °C for 30 s, followed by 30 cycles at 98 °C for 10 s, 62 °C for 20 s, and 72 °C for 15 s, and a final extension at 72 °C for 5 min. Five microliters of the PCR products were run on a 1.5 % agarose gel to verify correct amplification. The PCR products were purified using 10 U of Exonuclease I and 1 U of FastAP^TM^ Thermosensitive Alkaline Phosphatase (Fermentas GmbH, Germany) for 15 min at 37 °C, followed by enzyme inactivation for 15 min at 85 °C.

The purified PCR products were then sequenced with the same primers used in amplification using Big Dye Terminator (v3.1) Cycle Sequencing Kit (Applied Biosystems). Briefly, each 20 μl sequencing reaction mixture contained 2 μl of PCR amplicon, 0.16 μM of either forward or reverse PCR primer, 0.5 μl of BigDye Ready Reaction Mix, and 1 X sequencing buffer. The sequencing reaction conditions were as follows: 30 cycles of denaturing at 96 °C for 10 s, annealing at 50 °C for 5 s, and extension at 60 °C for 4 min. The sequencing products were purified using ethanol/EDTA/sodium acetate precipitation. Sequencing was performed on an ABI 3130xl 16-capillary automated genetic analyzer.

### MLST data treatment

The raw sequences were manually inspected and corrected using Sequencher 5.0.1 (Gene Codes, Ann Arbor, MI). The consensus sequence for each gene was determined by alignment of the forward and reverse sequences using BioEdit Sequence Alignment Editor Version 7.0.5.3 [31] (http://www.mbio.ncsu.edu/BioEdit/bioedit.html). The consensus sequences were aligned with Clustal W implemented in MEGA v5.2 [32]. For each housekeeping gene, allele numbers were assigned to unique sequences using the non-redundant databases (NRDB) program (http://www.mlst.net/). For each bacterial isolate, an allelic profile was determined as the combination of alleles at the six loci selected for final analyses. Finally, strains with an identical allelic profile were assigned to the same sequence type (ST) (Additional file 1).

### Phylogenetic analysis (MLSA)

A Neighbour-Joining dendrogram was constructed using individual and concatenated sequences of the six genes that were selected for final analyses. For comparison, the *F. columnare* type strain NCIMB 2248^T^, ATCC49512 and strain JIP39/87 were also included in the phylogenetic analysis. F*. psychrophilum* JIP 02/86 strain from France was used as an outgroup strain. MEGA v5.2 [32] was used to evaluate the model for nucleotide substitutions at each protein-coding locus and to construct a phylogenetic tree. The best model, having the lowest Bayesian Information Criterion (BIC) value, was used to generate the Neighbour-Joining tree based on 1000 replicates. The Tamura-Nei model plus a gamma distribution (T93 + G) model was used to infer the dendrogram for the concatenated sequences. A Chi-square test was employed to evaluate whether the presence of MLSA phylogenetic clusters is explained by regional (north and south) separation. Logistic regression was used to explore trends in outbreak likelihood over time for each clade. Moreover, the distribution of MLSA clusters (genotypes) across sampling origin (water or fish) were determined using Pearson's chi-square statistic, or, where sample sizes were small, Fisher's exact test. A P-value less than or equal to 0.05 was defined as statistically significant.

### Computational analysis

Analysis of DNA sequence variation of the housekeeping gene loci, including the number of alleles for each locus, GC (guanine + cytosine) content, the number of segregating sites (S), allelic diversity, and the nucleotide diversity (Pi), was carried out using DNAsp genetic software v5.10.01 [33]. MEGA v5.2 was used to perform Tajima’s D test [34]. The START 2 package [35] was used to determine the ratios of non-synonymous to synonymous substitutions (dN/dS) for each locus. The range of intraspecific sequence similarity (%) for each gene was resolved using BioEdit program [31].

### Population genetic and recombination analyses

Evidence for clonal or recombining population structures can be estimated by assessing the level of linkage between alleles at different loci. To test the null hypothesis, i.e. whether alleles of the six MLST loci used in the analyses are independent (linkage equilibrium), the IAS (standardized index of association) values were calculated with the START2 program. The test was performed first for the entire data set of 83 isolates and then for only eight STs to avoid biased results due to unequal sample sizes in different STs. IAS values significantly different from 0 indicate that a population is clonal (linkage disequilibrium), whereas non-significant values indicate a recombining population structure [36, 37].

The concatenated sequence data (for the six core genes) were formatted using xmfa2struct (http://www.xavierdidelot.xtreemhost.com/ClonalFrame.htm). Bayesian approach model with STRUCTURE version 2.3 [38] was used to determine the levels of inter-lineage recombination and the underlying population structure present in our data. Ten independent runs were performed for each value of the number of ancestral populations (*K)* ranging from 2 to 6. STRUCTURE was run for 500,000 Markov Chain Monte Carlo (MCMC) iterations following 250,000 burn-in iterations. The linkage model that reconstructs ancestral populations from DNA polymorphism data was used. The STRUCTURE Harvester [39], which implements the Evanno method [40], was used to identify the most probable groups (*K*) that best fit the data.

In order to estimate the mutation and recombination rates in *F. columnare*, we also performed recombination analysis using ClonalFrame v1.1 [41]. Three independent runs of ClonalFrame were performed, each consisting of 500,000 MCMC iterations and 250,000 burn-in iterations. ClonalFrame was also used to compare independent runs by the method of Gelman and Rubin [42]. ClonalFrame estimates ρ/θ, which measures the relative frequency of occurrence of recombination and mutation in the history of the lineage, and r/m which measures the relative impact of recombination and mutation in the genetic diversification of the lineage. The values of ρ/θ and r/m for all 83 isolates and for each lineage were calculated by extracting the numbers of mutation events, recombination events, and substitutions introduced by recombination from the ClonalFrame output.

## Results

### MLST

Thirteen housekeeping genes were successfully amplified for the 83 *F. columnare* isolates from Finland and for both the type strain NCIMB 2248^T^ and the isolate JIP39/87. The housekeeping genes *ftsQ, glyA, murG, recA, fumC, 16S rDNA*, and *rplB* showed identical sequences among all 83 Finnish strains and were excluded from further analyses. Six remaining housekeeping genes, *trpB*, *tuf*, *atpA*, *rpoD*, *gyrB* and *dnaK* were selected to develop the MLST/MLSA scheme. The range of intraspecific sequence similarity (%) calculated for each gene showed that *trpB* had the highest level of sequence polymorphism between the strains (94.5 % similarity), followed by *rpoD* (97.4 %). Whereas the lowest levels of inter-strain sequence variation were found for *tuf* (99.5 %), *atpA* (99.5 %), gyrB (99.6 %), and *dnaK* (99.8 %). The GC content for individual genes exhibited very little variation between the strains (≤ ±0.5 %) (Table 1). The average overall GC content for the six genes in all 83 Finnish strains was 39 %, slightly higher than the overall GC content (32.5 %) for the *F. columnare* ATCC 49512 genome [17].

**Table 1:** Characteristics of the six loci used in the analyses for the 83 *F. columnare* strains

Locus	Fragment size(bp)	No. of alleles	Allelic diversity	GC	S	dN/dS	∏	Tajima’s D
trpB	626	5	0.72	0.441	49	0.046	0.0283	2.45^a^
rpoD	386	3	0.53	0.348	10	0.000	0.0117	3.38^a^
tuf	608	4	0.29	0.399	3	0.126	0.0005	−0.85^b^
atpA	645	4	0.56	0.425	3	0.000	0.0015	0.52^b.^
dnak	643	2	0.27	0.362	1	0.000	0.0004	0.43^b.^
gyrB	598	3	0.37	0.369	2	0.000	0.0006	−0.07^b.^
Concatenated	3509	9	0.74	0.390	68	-	-	-
Average	-	-	0.4604	-	-	-	-	-

*GC* Guanine + Cytosine content; *S* number of segregating sites; *dN/dS* the ratio of non-synonymous to synonymous substitutions; *∏* nucleotide diversity; *Tajima’s D* neutrality test values

^a^statistically significant, and ^b^non-significant value

Using the MLST method, the *F. columnare* strains were divided into 8 different sequence types (STs). The number of alleles at each locus ranged from 2 (*dnaK)* to 5 (*trpB*), and the number of variable sites varied between 1 (*dnaK*) and 49 (*trpB*) (Table 1). No insertions or deletions were detected within the loci. The allelic diversity ranged from 0.27 for *dnaK* to 0.72 for *trpB*, with an average value of 0.46 (Table 1). The average pairwise nucleotide diversity π for all genes was 4.2 %, with π-values for individual loci ranging from 0.0004 for *dnaK* to 0.0283 for *trpB* (Table 1). Tajima’s D values for loci *tuf*, *atpA*, *dnaK*, and *gyrB* were nonsignificant, supporting neutrality of the alleles of these genes (i.e., evolution by random processes) (Table 1). In contrast, *trpB* and *rpoD* both showed a significantly positive value for Tajima's D, indicating a decrease in population size, balancing selection [43], or subdivision of the population [44].

### Phylogenetic analysis (MLSA)

**Fig. 2:**
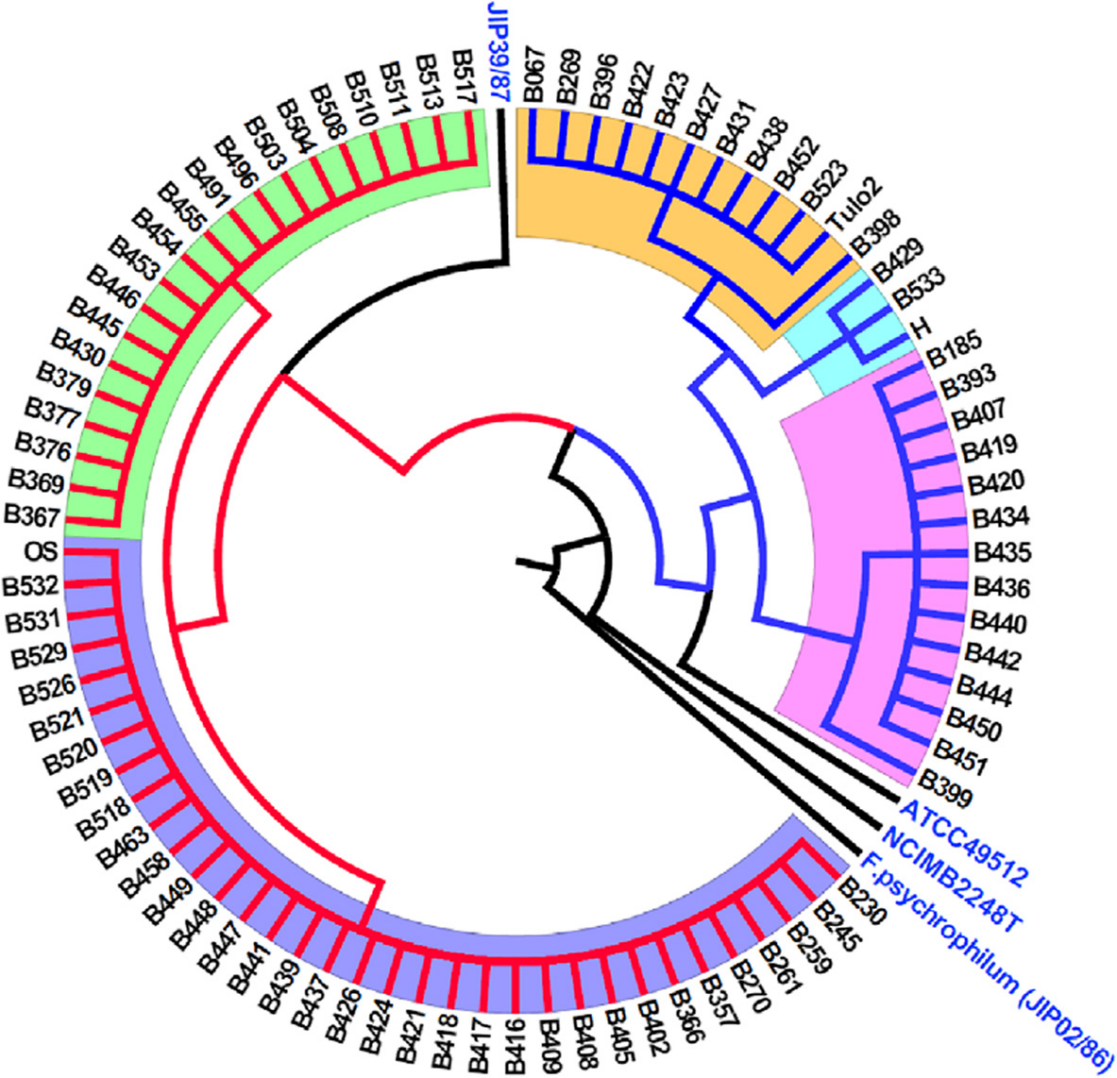
Phylogenetic tree of *F. columnare* strains based on a concatenated 6-housekeeping gene (*trpB, tuf, atpA, rpoD, gyrB and dnaK*). Strict consensus Neighbour Joining tree for the 83 *F. columnare* strains from Finland and three other strains, including the type strain NCIMB 2248^T^, ATCC 49512, and strain JIP39/87. The numbers at the nodes represent levels (%) of bootstrap support from 1000 resampled datasets. The sequence of the *Flavobacterium psychrophilum* (JIP 02/86) strain from France was used as an out-group. MLSA clusters (designated in accordance with the corresponding ARISA genotypes) C, E, A, H, and G are marked with purple, green, orange, light blue, and pink, respectively. Branches from Finnish lineage I strains are coloured red, those from lineage II are coloured blue, and those from other countries are coloured black

In total, 3,509 bp of sequence was analyzed for each strain. The phylogenetic tree based on the concatenated sequence shows two major lineages (I and II) which can be further divided into five MLSA clusters (Fig. 2 and Additional file 3). Both lineages are strongly supported by high bootstrap values. The clusters within lineages associated uniformly with the ARISA genotypes of the strains (for more information, see Additional file 4). Therefore, we decided to designate the MLSA clusters in accordance with the corresponding ARISA genotypes [8]. Lineage I includes two clusters, corresponding to ARISA genotypes C and E. Cluster C (ST2) contains 36 strains isolated from six locations: NorthA, NorthB, NorthC, SouthA, SouthC, and SouthD (see Figs. 1 and 2). Cluster E (STs 4 and 8) includes 20 strains isolated from SouthC. Lineage II includes three clusters, corresponding to ARISA genotypes G, A and H. Cluster G (STs 1 and 7) contains strains isolated from NorthB, NorthC, SouthB and SouthC. Cluster A (STs 3 and 6) includes strains isolated from NorthB, NorthD, SouthB, and SouthC. Cluster H (ST5) contains three strains isolated form NorthB and SouthC. Statistical analysis showed that there was no significant regional (north and south) clustering of *F. columnare* in Finland, but the occurrence of cluster E isolates was more frequent in more recent years (Table 2). No other trends were apparent over the study period 2003–2012 (Table 2). Cluster H strains (total of 3 isolates) were only found in years 2003 and 2012, which most likely results from random sampling of a very rare genotype, rather than any systematic reasons. Furthermore, no association was found between MLSA clusters and the sampling origin including water and fish species (P = 0.52, Additional file 1).

**Table 2:** Proportion of isolates of *F. columnare* from each region (south and north) and year of isolation within the MLSA clusters (%)

MLSA clusters	No. of isolates	North	South	P value	2003	2005	2006	2007	2008	2009	2010	2011	2012	P value
C	34	58.8	41.2	0.182	0	0	23.5	14.7	8.8	20.6	17.6	2.9	11.8	0.88
E	20	0	100	-	5.0	0	0	10.0	15.0	0	25.0	5.0	40.0	0.003^*^
G	14	57.1	42.9	0.593	0	14.3	7.1	35.7	14.3	7.1	21.4	0	0	0.145
H	3	33.3	66.7	0.564	66.7	0	0	0	0	0	0	0	33.3	-^a^
A	12	50	50	1.00	9.1	0	18.2	27.3	0	27.3	18.2	0	0	0.118

Observations for regional sources were compared by nonparametric chi-square test (* = *P* < 0.05). Observations for year of isolation were tested with logistic regression where year was fitted as a continuous covariate

^a^Due to the small number of cluster H isolates in our dataset (*n* = 3), we did not include cluster H in logistic regression

The phylogenetic and Bayesian analyses clearly indicated that *F. columnare* strains are genetically distinct from *F. psychrophilum.* In addition, the early-branching type strain (NCIMB 2248^T^) from the USA appears to be highly divergent from the Finnish *F. columnare* strains. However, the reference strains ATCC 49512 and JIP 39/87, both from France, were phylogenetically close to isolates from Finland and clustered with the Finnish strains.

### Population genetic and recombination analysis

**Fig. 3:**
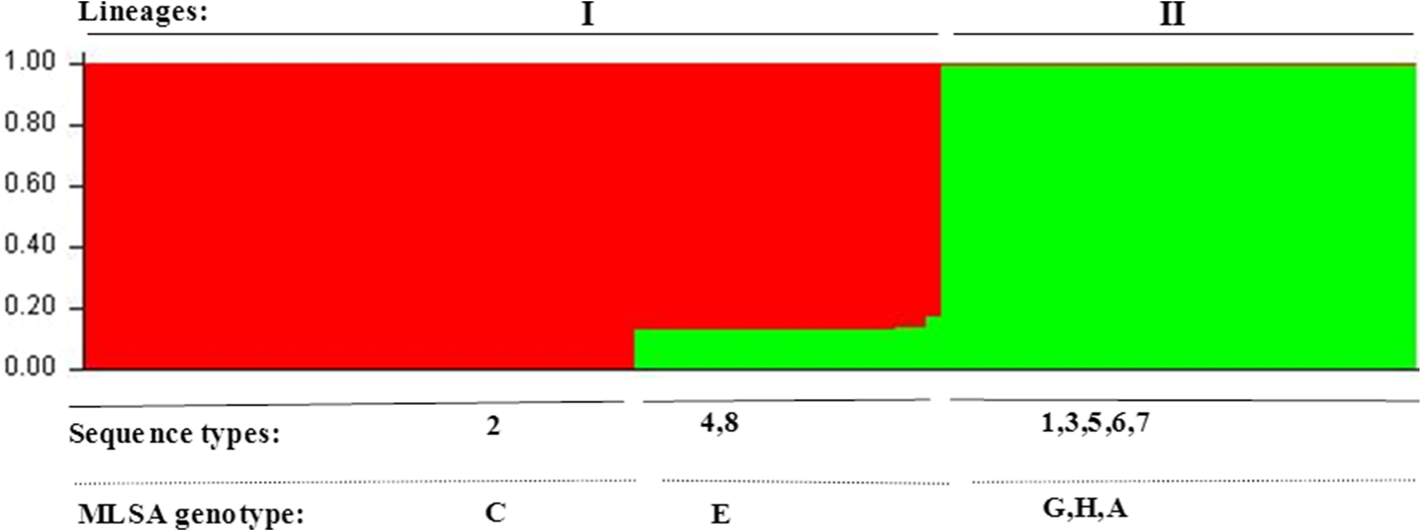
Estimated *F. columnare* population structure using STRUCTURE version 2.3. Ancestral population size of 2 (K = 2) was chosen as the best fit for the current data. Upper labels indicate the ancestral populations (lineages); sequence types and MLSA clusters of the isolates are listed at the bottom

Results from the ClonalFrame analysis (based on both the 83 Finnish isolates and eight STs) showed that mutations have occurred approximately seven times more frequently than recombination (ρ/θ ≈ 0.14; with 95 % confidence interval of 0.03-0.9). However, recombination has had a significant effect in the evolutionary process as shown by the r/m value of approximately three (with 95 % confidence interval of 0.44-6.6). This indicates that even though recombination has been less frequent than mutation, each recombination event has introduced almost three times more substitutions than mutation. According to the results extracted from the ClonalFrame output, the role of recombination seems to be uneven across the two MLSA lineages. The relative frequency of occurrence of recombination *versus* mutation (ρ/θ) was 0.2 for lineage I, and 0.08 for lineage II. Whereas the relative impact of recombination *versus* point mutation (r/m) was 3.14 for lineage I and 1.43 for lineage II. However, even in case of high recombination rate, analysis of all 83 isolates yielded an IAS (standardized index of association) value of 0.292 (*P* < 0.001), indicating persistence of identical clonally expanded sequence types over sampling years. Moreover, IAS values calculated separately for lineages I and II were also significantly different from zero (0.81 and 0.65, respectively, *P* < 0.001 for both). When only one representative of each sequence type was included, the overall IAS value was decreased to 0.198, but remained significant (P = 0.03), meaning that recombination was not sufficient to break down linkage disequilibrium. The analysis with STRUCTURE detected two subpopulations (best K = 2), denoted as lineages I and II (Fig. 3). Lineage II forms a genetically homogeneous group. However, lineage I represent a genetically heterogeneous group with genetic material imported from lineage II.

## Discussion

In this paper, we report the results of the first MLST/MLSA scheme developed to determine the genetic variability and population genetic structure of the *F. columnare* strains isolated from different locations in Finland. According to classification based on 16 s rDNA-RFLP analysis, all Finnish isolates studied thus far belong to genomovar I [8] (see Additional file 5). This is likely explained by the fact that strains from genomovars II and III have higher temperature requirements and thus may not be able to tolerate colder temperatures in Finland; it has been shown that these genomovars thrive at higher temperatures but not at 15 °C [10]. Therefore, because we focus here on the Finnish population, isolates from genomovars II and III were not included in the study. Our results demonstrated that Finnish *F. columnare* strains were separated into different STs (by MLST) and phylogenetic clusters (by MLSA) irrespective of the geographic origin, host species or year of isolation. Previous studies have reported a similar lack of relationship between genotypes and isolation source including both host and geographical origin. Based on three genotyping methods including rDNA-RFLP, ISR and AFLP, Arias *et al.* [14] analysed 30 *F. columnare* isolates from four countries and found that these strains did not cluster according to host species or geographic origin. Similarly, an analysis of ten *F. columnare* isolates originating from cold and warm waters using RFLP analysis showed no relationship between geographic origin and genomic types [4]. One possible explanation for the lack of association between STs and their geographic origin (i.e. genetically similar populations occur in geographically distinct areas) is that the transportation of fish stocks or equipment between fish farms and natural exchanges such as wild bird could contribute to the spread of *F. columnare* strains between regions [45].

The six loci used in the analyses (*trpB*, *tuf*, *atpA*, *rpoD*, *gyrB*, *dnaK*) were successfully amplified and sequenced for all isolates. Based on MLST statistical analyses and concatenated phylogenetic analyses, all 83 isolates were grouped into eight STs and two major lineages (I and II) that were further assigned into five clusters (C, E, A, H, and G) (Fig. 1). Overall, the isolates were commonly designated to cluster C while cluster H strains were the rarest ones. We found that the environmental isolates of *F. columnare* are phylogenetically clustered with the epidemic strains, indicating that the environmental population may be the source of epidemic strains and *vice versa*. Non-synonymous base substitutions in gene sequences were rare (*dN/dS* < 1) (Table 1), indicating purifying selection against amino acid changes. This verifies that the identified sequence variability is selectively neutral at the protein level, making the sequences good candidates for multilocus sequence typing and analysis. However, an excess of polymorphisms in the *trpB* and *rpoD* sequences can also indicate balancing selection, supported also by the positive Tajima’s D values. This finding is consistent with two clearly distinct lineages in the Finnish sample of *F. columnare*. It is also possible that the differences in *trpB* and *rpoD* are caused by both purifying selection (limiting the amino acid changes due to functional constraints on housekeeping genes), and potential balancing selection (maintaining the genetic polymorphism within a population).

The individual trees based on *trpB* and *rpoD* were found to provide higher phylogenetic resolution than corresponding *tuf*, *atpA*, *dnaK*, and *gyrB* gene sequences (Additional file 1). Although cluster H strains could only be resolved by using the concatenated sequences of all six housekeeping gene sequences, the tree constructed from the *trpB* gene alone could identify four major clusters (A, C, E, and G,) resolved by MLSA analysis. Moreover, to determine whether the *trpB* gene used in the concatenated sequence influenced tree topology, it was compared to the tree constructed on five MLSA gene sequences (i.e. excluding *trpB* gene). Adding the *trpB* sequence to the MLSA-concatenated sequence (*rpoD, dnaK, tuf, gyrB,* and *atpA)* thus had minor effect on the clustering of strains. Furthermore, we found that only one strain, B399, had discordant *trpB*-based identification (Additional files 3 and 6). Thereby, we propose that the *trpB* gene can serve as a reliable, cost-effective, and quick molecular marker to differentiate closely related *F. columnare* strains.

Significant linkage disequilibrium between the *F. columnare* MLST alleles suggests a non-random distribution of alleles and a clonal population structure where diversity increases by the accumulation of point mutations. Indeed, recovering identical or similar STs from different geographic origins suggests that *F. columnare* strains may undergo clonal expansion. This conclusion is also supported by the observation that the same genotypes are repeatedly isolated over the years. On the other hand, the low IAS values (although significant) suggest that recombination may have occurred among these strains, but not frequently enough to completely remove linkage disequilibrium. Finding linkage disequilibrium between the alleles is not surprising, considering that an allele must change at least 20 times more frequently by recombination than by point mutation to achieve random assortment within a bacterial population [37]. Based on both STRUCTURE and ClonalFrame analyses, however, recombination events have occurred both within and across lineages. Considering both the relative frequency of occurrence of recombination and mutation (ρ/θ ≈ 0.14) and the relative effect of recombination and mutation in genetic diversification (r/m ≈ 3), the Finnish *F. columnare* strains seem to have a moderate recombination rate. The observed genetic exchange and recombination within and across the two lineages is irreconcilable with a strictly clonal population structure. Therefore, we suggest that the *F. columnare* population structure in Finland follows an epidemic population structure where there is a background level of frequent recombination with consecutive clonal expansion of one or a few fit genotypes [46], similar to that of other bacterial species such as *Escherichia coli* [47], *Vibrio parahaemolyticus* [48] and *F. psychrophilum* [49]*.* Compared with other fish pathogens, the estimated recombination rate for the Finnish *F. columnare* strains seems to be slightly higher than in *Tenacibaculum maritimum* (∼2.4:1) [50], and is clearly lower than in the highly recombinogenic bacterium *F. psychrophilum* (∼26:1) [51]. However, as our data set consists of local samples, we cannot estimate the recombination rate of *F. columnare* at a global (species) level.

Our results further demonstrate that lineage I displays more evidence of recombination than lineage II (r/m ≈ 3.14 and 1.43, respectively). We also show that only lineage I seems to exhibit mixed ancestries (lower than 20 %) suggesting that limited, unidirectional inter-lineage admixture has taken place (Fig. 3). Such infrequent recombination between lineages is also consistent with our results from the phylogenetic analysis, indicating divergence of the two lineages (I and II). A low level of inter-lineage recombination, even among sympatric strains, coupled with high levels of intra-lineage recombination suggests that natural barriers could prevent recombination across the two lineages. Since the statistical analysis indicates that the clustering of the lineages is not caused by geography, the barrier against inter-lineage gene transfer may be caused by other, yet undefined, factors.

## Conclusions

Our MLST/MLSA scheme data demonstrate that both recombination and clonality play a role in shaping the population structure of *F. columnare* in Finland. The population structure of the Finnish *F. columnare* is probably semi-clonal which diversifies with moderate, but variable, recombination rate. Limited association of genotypes with geography or year of isolation indicates that columnaris outbreaks in Finland are caused by continuous co-circulation of *F. columnare* strains. Furthermore, recovering identical genotypes from both fish and from the environment confirms that environmentally persistent strains are also epidemiologically important.

## Availability of supporting data

DNA sequences of all STs have been submitted to the European genetic database EMBL under the following accession numbers: *trpB* (LN624115, LN624121, LN624122, LN624123, and LN624124), *rpoD* (LN624116, LN624125, and LN624126), *atpA* (LN624118, LN624133, LN624134, and LN624135), *tuf* (LN624120, LN624130, LN624131, and LN624132), *gyrB* (LN624119, LN624128, and LN624129), *dnaK* (LN624117, LN624127).

## Additional files

Additional file 1 Site, year of isolation, source of isolation (fish or water), location of isolation, host species, sequence type (ST), and allelic profile data for the 83 F. columnare strains from Finland analyzed by MLST. (DOCX 46 kb) Additional file 2 Target genes and primers for the housekeeping genes of *F. columnare.* The loci used for the MLST/MLSA scheme are shown in bold font. Length refers to the length of the target sequence. * Reference for 16S rDNA primers [52]. (DOCX 20 kb) Additional file 3 Neighbor Joining phylogenetic trees based on the individual sequences of six MLST loci (*trpB, rpoD, gyrB, dnaK, atpA,*and *tuf*). MEGA v5.2 was used to evaluate the models for nucleotide substitution for each protein-coding locus and to construct the phylogenetic trees for Finnish *F. columnare* strains. The *F. columnare* type strain NCIMB 2248^T^ isolated in the USA and two reference strains JIP39/87 and ATCC49512, both isolated in France, were also included in the phylogenetic analysis. The best model indicated by the lowest Bayesian Information Criterion (BIC) value was used to generate the Neighbor-Joining tree based on 1000 replicates. The T92 model was selected for *dnaK*, *tuf,* and *gyrB*, while T92 + G was selected for both *trpB* and *rpoD* and JC model was selected for *atpA*. Strains corresponding to lineage I (cluster C and E) are coloured red and strains corresponding to lineage II (cluster G, A and H) are in blue. Other sequences assigned to reference strains are in black. Few strains representative of each genotype were used for tree construction (identical sequences removed for clarity of representation). (PDF 120 kb) Additional file 4 The fluorescence peak profiles for the ARISA genotypes analysed with ABI Prism 3130xl Genetic Analyser and the GeneMapper v.5.0 software (Applied Biosystems, Carlsbad, California, USA). For the 83 strains isolated from Finland, we also determined the ARISA (automated ribosomal intergenic spacer analysis) genotypes following the procedure described by Suomalainen et al. [8]. However, the previously published method was modified so that ABI Prism 3130xl Genetic Analyser is used instead of LI-COR 4200 automatic sequencer The analysis revealed that ARISA genotypes associate uniformly with the clusters from the MLSA scheme. Briefly, the PCR reaction mixture (total volume 10 ul) contained 1X DreamTaq Buffer (Thermo Scientific), 0.2 mM dNTPs (Thermo Scientific), 0.5 μM reverse primer (23Sr, 5’-GGGTTBCCCCATTCRG-3), 0.44 μM forward primer (rD1f, 5’-GGCTGGATCACCTCCTT-3’), 0.06 μM 6-carboxyfluorescein (6-FAM) labelled forward primer (rD1f), 1 U of DreamTaq DNA Polymerase (Thermo Scientific) and 1 μl of template DNA. The PCR reactions were carried out using Bio-Rad C1000 or S1000 thermal cyclers (Bio-Rad Laboratories, Hercules, CA, USA). The thermo-cycling conditions included initial denaturation at 95 °C for 2 min followed by 30 cycles of amplification (94 °C for 30 s, 52 °C for 30 s, 72 °C for 3 min) and a final extension at 72 °C for 15 min. The PCR products were denatured with formamide and GeneScanTM 1200 LIZ Size Standard was added. Products were separated with an ABI Prism 3130xl Genetic Analyser, and visualized with GeneMapper v.5.0 software (all Applied Biosystems, Carlsbad, California, USA). Based on the fluorescence peak profiles and using the strains from Suomalainen et al. [8] as positive controls, the 87 *F. columnare* strains were designated into ARISA genotypes A to H [8]. (PDF 222 kb) Additional file 5 Phylogenetic tree based on the 16 s rDNA sequence data obtained from the representatives of Finnish *F .columnare* genotypes (A-H) studied in this study and other *F .columnare* sequences obtained from the GenBank. The tree was constructed by a UPGMA clustering method with a resampling of 1,000 bootstrap replicates and the Jukes Cantor model. Two strains representative of each ARISA genotype/MLSA cluster studied in this study were used for tree construction (identical sequences removed for clarity of representation). (PDF 191 kb) Additional file 6 The concatenated tree based on six housekeeping MLSA genes (including *trpB;* right) and five MLSA gene sequences (*rpoD, dnaK, tuf, gyrB, atpA*; left). The arrow shows disagreement concerning the position of strain G2 (B399) between the two trees. (PDF 77 kb)

## Abbreviations

MLSA: Multilocus sequence analysis
MLST: Multilocus sequence typing
ATCC: American type culture collection
16S rDNA-RFLP: 16S rDNA by restriction-fragment length polymorphism
SSCP: Single-strand conformation polymorphism
AFLP: Amplified fragment length polymorphism
PFGE: Pulsed-field gel electrophoresis
ARISA: Automated ribosomal intergenic spacer analysis
ISR-SSCP: Internal spacer region-single strand conformation polymorphism analysis
MCMC: Markov Chain Monte Carlo
IAS: Standardized index of association
dn/ds: the ratios of non-synonymous to synonymous substitutions
T93 + G: Tamura-Nei model and a gamma distribution
BIC: Bayesian Information Criterion
GC: Guanine + Cytosine content
S: the number of segregating sites
