# Supplementary material for: A multilocus sequence analysis scheme for characterization of Flavobacterium columnare isolates
Source: BMC Microbiol. 2015 Oct 30;15:243. doi: 10.1186/s12866-015-0576-4 (PMC4628280; doi:10.1186/s12866-015-0576-4)
Supplement: Additional file 1 — Site, year of isolation, source of isolation (fish or water), location of isolation, host species, sequence type (ST), and allelic profile data for the 83 F. columnare strains from Finland analyzed by MLST. (DOCX 46 kb) [file 12866_2015_576_MOESM1_ESM.docx]

**Additional File 1.** Site , year of isolation, source of isolation (fish or water), location of isolation, host species, sequence type (ST), and allelic profile data for the 83 *F. columnare* strains from Finland analyzed by MLST.

| Strain | Site | Year | Source | Location | Host | ST | *trpB* | *rpoD* | *tuf* | *atpA* | *dnaK* | *gyrB* |
| --- | --- | --- | --- | --- | --- | --- | --- | --- | --- | --- | --- | --- |
| B531 | NorthA | 2012 | Fish | northern | brown trout (*Salmo trutta*) | 2 | 2 | 2 | 2 | 1 | 1 | 1 |
| B532 | NorthA | 2012 | Fish | northern | brown trout (*Salmo trutta*) | 2 | 2 | 2 | 2 | 1 | 1 | 1 |
| B529 | NorthC | 2012 | Fish | northern | Atlantic salmon (*Salmo salar)* | 2 | 2 | 2 | 2 | 1 | 1 | 1 |
| B526 | NorthC | 2012 | Fish | northern | Atlantic salmon (*Salmo salar*) | 2 | 2 | 2 | 2 | 1 | 1 | 1 |
| B521 | NorthA | 2006 | Fish | northern | Atlantic salmon (*Salmo salar*) | 2 | 2 | 2 | 2 | 1 | 1 | 1 |
| B520 | NorthA | 2006 | Fish | northern | Atlantic salmon (*Salmo salar*) | 2 | 2 | 2 | 2 | 1 | 1 | 1 |
| B519 | NorthA | 2006 | Fish | northern | brown trout (*Salmo trutta*) | 2 | 2 | 2 | 2 | 1 | 1 | 1 |
| B518 | NorthB | 2006 | Fish | northern | Atlantic salmon (*Salmo salar*) | 2 | 2 | 2 | 2 | 1 | 1 | 1 |
| B463 | SouthD | 2011 | Fish | southern | rainbow trout (*Oncorhynchus mykiss*) | 2 | 2 | 2 | 2 | 1 | 1 | 1 |
| B458 | NorthB | 2009 | Fish | northern | brown trout (*Salmo trutta*) | 2 | 2 | 2 | 2 | 1 | 1 | 1 |
| B449 | SouthA | 2007 | Water | southern | Water | 2 | 2 | 2 | 2 | 1 | 1 | 1 |
| B448 | SouthA | 2007 | Water | southern | Water | 2 | 2 | 2 | 2 | 1 | 1 | 1 |
| B447 | SouthC | 2007 | Water | southern | Water | 2 | 2 | 2 | 2 | 1 | 1 | 1 |
| B441 | NorthB | 2007 | Fish | northern | Atlantic salmon (*Salmo salar*) | 2 | 2 | 2 | 2 | 1 | 1 | 1 |
| B439 | SouthD | 2006 | Fish | southern | rainbow trout (*Oncorhynchus mykiss*) | 2 | 2 | 2 | 2 | 1 | 1 | 1 |
| B437 | NorthB | 2006 | Fish | northern | brown trout (*Salmo trutta*) | 2 | 2 | 2 | 2 | 1 | 1 | 1 |
| B426 | NorthB | 2006 | Fish | northern | Atlantic salmon (*Salmo salar)* | 2 | 2 | 2 | 2 | 1 | 1 | 1 |
| B424 | NorthA | 2007 | Fish | northern | brown trout (*Salmo trutta*) | 2 | 2 | 2 | 2 | 1 | 1 | 1 |
| B421 | NorthB | 2008 | Fish | northern | Atlantic salmon (*Salmo salar*) | 2 | 2 | 2 | 2 | 1 | 1 | 1 |
| B418 | NorthA | 2009 | Fish | northern | brown trout (*Salmo trutta*) | 2 | 2 | 2 | 2 | 1 | 1 | 1 |
| B417 | NorthA | 2008 | Fish | northern | Atlantic salmon (*Salmo salar*) | 2 | 2 | 2 | 2 | 1 | 1 | 1 |
| B416 | NorthA | 2008 | Fish | northern | brown trout (*Salmo trutta)* | 2 | 2 | 2 | 2 | 1 | 1 | 1 |
| B409 | NorthA | 2010 | Fish | northern | sea trout (*Salmo trutta trutta*) | 2 | 2 | 2 | 2 | 1 | 1 | 1 |
| B408 | SouthC | 2010 | Water | southern | Water | 2 | 2 | 2 | 2 | 1 | 1 | 1 |
| B405 | SouthA | 2010 | Water | southern | Water | 2 | 2 | 2 | 2 | 1 | 1 | 1 |
| B402 | SouthC | 2010 | Fish | southern | European whitefish(*Coregonus lavaretus*) | 2 | 2 | 2 | 2 | 1 | 1 | 1 |
| B366 | SouthC | 2010 | Water | southern | Water | 2 | 2 | 2 | 2 | 1 | 1 | 1 |
| B357 | SouthC | 2010 | Water | southern | Water | 2 | 2 | 2 | 2 | 1 | 1 | 1 |
| B270 | NorthB | 2009 | Water | northern | Water | 2 | 2 | 2 | 2 | 1 | 1 | 1 |
| B261 | SouthC | 2009 | Water | southern | Water | 2 | 2 | 2 | 2 | 1 | 1 | 1 |
| B259 | SouthC | 2009 | Water | southern | Water | 2 | 2 | 2 | 2 | 1 | 1 | 1 |
| B245 | SouthC | 2009 | Water | southern | Water | 2 | 2 | 2 | 2 | 1 | 1 | 1 |
| B230 | SouthC | 2009 | Water | southern | Water | 2 | 2 | 2 | 2 | 1 | 1 | 1 |
| Os06 | NorthC | 2006 | Fish | northern | Atlantic salmon (*Salmo salar*) | 2 | 2 | 2 | 2 | 1 | 1 | 1 |
| B367 | SouthC | 2010 | Water | southern | Water | 4 | 4 | 2 | 2 | 3 | 1 | 2 |
| B369 | SouthC | 2010 | Fish | southern | brown trout (*Salmo trutta*) | 4 | 4 | 2 | 2 | 3 | 1 | 2 |
| B376 | SouthC | 2010 | Water | southern | Water | 4 | 4 | 2 | 2 | 3 | 1 | 2 |
| B377 | SouthC | 2010 | Water | southern | Water | 4 | 4 | 2 | 2 | 3 | 1 | 2 |
| B379 | SouthC | 2010 | Water | southern | Water | 4 | 4 | 2 | 2 | 3 | 1 | 2 |
| B430 | SouthC | 2003 | Fish | southern | Pike perch (*Stizostedion lucioperca*) | 4 | 4 | 2 | 2 | 3 | 1 | 2 |
| B445 | SouthC | 2007 | Water | southern | Water | 4 | 4 | 2 | 2 | 3 | 1 | 2 |
| B453 | SouthC | 2008 | Fish | southern | rainbow trout (*Oncorhynchus mykiss*) | 4 | 4 | 2 | 2 | 3 | 1 | 2 |
| B454 | SouthC | 2008 | Fish | southern | rainbow trout (*Oncorhynchus mykiss*) | 4 | 4 | 2 | 2 | 3 | 1 | 2 |
| B455 | SouthC | 2008 | Fish | southern | rainbow trout (*Oncorhynchus mykiss*) | 4 | 4 | 2 | 2 | 3 | 1 | 2 |
| B491 | SouthC | 2012 | Water | southern | Water | 4 | 4 | 2 | 2 | 3 | 1 | 2 |
| B496 | SouthC | 2012 | Water | southern | Water | 4 | 4 | 2 | 2 | 3 | 1 | 2 |
| B503 | SouthC | 2012 | Fish | southern | rainbow trout (*Oncorhynchus mykiss*) | 4 | 4 | 2 | 2 | 3 | 1 | 2 |
| B504 | SouthC | 2012 | Fish | southern | rainbow trout (*Oncorhynchus mykiss*) | 4 | 4 | 2 | 2 | 3 | 1 | 2 |
| B508 | SouthC | 2012 | Fish | southern | rainbow trout (*Oncorhynchus mykiss*) | 4 | 4 | 2 | 2 | 3 | 1 | 2 |
| B510 | SouthC | 2012 | Fish | southern | rainbow trout (*Oncorhynchus mykiss*) | 4 | 4 | 2 | 2 | 3 | 1 | 2 |
| B511 | SouthC | 2012 | Fish | southern | rainbow trout (*Oncorhynchus mykiss*) | 4 | 4 | 2 | 2 | 3 | 1 | 2 |
| B513 | SouthC | 2012 | Fish | southern | rainbow trout (*Oncorhynchus mykiss*) | 4 | 4 | 2 | 2 | 3 | 1 | 2 |
| B446 | SouthC | 2007 | Water | southern | Water | 8 | 4 | 2 | 2 | 3 | 2 | 2 |
| B517 | SouthC | 2011 | Water | southern | Water | 4 | 4 | 2 | 2 | 3 | 1 | 2 |
| B450 | SouthB | 2007 | Fish | southern | Pike perch (*Stizostedion lucioperca*) | 1 | 1 | 1 | 1 | 1 | 1 | 1 |
| B451 | SouthB | 2007 | Fish | southern | Pike perch (*Stizostedion lucioperca*) | 1 | 1 | 1 | 1 | 1 | 1 | 1 |
| B444 | NorthC | 2007 | Fish | northern | Atlantic salmon (*Salmo salar*) | 1 | 1 | 1 | 1 | 1 | 1 | 1 |
| B442 | NorthC | 2007 | Fish | northern | Atlantic salmon (*Salmo salar*) | 1 | 1 | 1 | 1 | 1 | 1 | 1 |
| B440 | NorthB | 2007 | Fish | northern | Atlantic salmon (*Salmo salar*) | 1 | 1 | 1 | 1 | 1 | 1 | 1 |
| B436 | NorthC | 2006 | Fish | northern | Atlantic salmon (*Salmo salar*) | 1 | 1 | 1 | 1 | 1 | 1 | 1 |
| B435 | NorthC | 2005 | Fish | northern | Atlantic salmon (*Salmo salar*) | 1 | 1 | 1 | 1 | 1 | 1 | 1 |
| B434 | NorthC | 2005 | Fish | northern | Atlantic salmon (*Salmo salar*) | 1 | 1 | 1 | 1 | 1 | 1 | 1 |
| B420 | NorthB | 2009 | Fish | northern | Atlantic salmon (*Salmo salar*) | 1 | 1 | 1 | 1 | 1 | 1 | 1 |
| B419 | NorthB | 2009 | Fish | northern | Atlantic salmon (*Salmo salar*) | 1 | 1 | 1 | 1 | 1 | 1 | 1 |
| B407 | SouthC | 2010 | Water | southern | Water | 1 | 1 | 1 | 1 | 1 | 1 | 1 |
| B393 | SouthC | 2010 | Fish | southern | bream (*Abramis brama* ) | 1 | 1 | 1 | 1 | 1 | 1 | 1 |
| B185 | SouthB | 2009 | Water | southern | Water | 1 | 1 | 1 | 1 | 1 | 1 | 1 |
| B399 | SouthC | 2010 | Water | southern | Water | 7 | 1 | 3 | 3 | 1 | 1 | 1 |
| B429 | SouthC | 2003 | Fish | southern | Pike perch (*Stizostedion lucioperca*) | 5 | 3 | 3 | 2 | 1 | 1 | 1 |
| B533 | NorthB | 2012 | Fish | northern | Atlantic salmon (*Salmo salar*) | 5 | 3 | 3 | 2 | 1 | 1 | 1 |
| H | SouthC | 2003 | Fish | southern | rainbow trout (*Oncorhynchus mykiss*) | 5 | 3 | 3 | 2 | 1 | 1 | 1 |
| B398 | SouthC | 2010 | Water | southern | Water | 6 | 3 | 3 | 2 | 2 | 1 | 1 |
| B269 | NorthB | 2009 | Water | northern | Water | 3 | 3 | 3 | 2 | 2 | 2 | 1 |
| B396 | SouthC | 2010 | Water | southern | Water | 3 | 3 | 3 | 2 | 2 | 2 | 1 |
| B422 | NorthD | 2009 | Fish | northern | sea trout (*Salmo trutta trutta*) | 3 | 3 | 3 | 2 | 2 | 2 | 1 |
| B423 | NorthD | 2009 | Fish | northern | lake trout (*Salmo trutta lacustris*) | 3 | 3 | 3 | 2 | 2 | 2 | 1 |
| B427 | NorthD | 2006 | Fish | northern | brown trout (*Salmo trutta*) | 3 | 3 | 3 | 2 | 2 | 2 | 1 |
| B431 | SouthB | 2003 | Fish | southern | grayling *Thymallus thymallus* | 3 | 3 | 3 | 2 | 2 | 2 | 1 |
| B438 | NorthD | 2006 | Fish | northern | rainbow trout (*Oncorhynchus mykiss*) | 3 | 3 | 3 | 2 | 2 | 2 | 1 |
| B452 | SouthB | 2007 | Fish | southern | Pike perch (*Stizostedion lucioperca)* | 3 | 3 | 3 | 2 | 2 | 2 | 1 |
| B523 | NorthD | 2007 | Fish | northern | brown trout (*Salmo trutta*) | 3 | 3 | 3 | 2 | 2 | 2 | 1 |
| B067 | SouthB | 2007 | Fish | southern | brown trout (*Salmo trutta*) | 3 | 3 | 3 | 2 | 2 | 2 | 1 |
| tulo2 | SouthC | 2010 | Water | southern | Water | 3 | 3 | 3 | 2 | 2 | 2 | 1 |
