# Supplementary material for: A multilocus sequence analysis scheme for characterization of Flavobacterium columnare isolates
Source: BMC Microbiol. 2015 Oct 30;15:243. doi: 10.1186/s12866-015-0576-4 (PMC4628280; doi:10.1186/s12866-015-0576-4)
Supplement: Additional file 2 — Target genes and primers for the housekeeping genes of F. columnare. The loci used for the MLST/MLSA scheme are shown in bold font. Length refers to the length of the target sequence. * Reference for 16S rDNA primers [52]. (DOCX 20 kb) [file 12866_2015_576_MOESM2_ESM.docx]

**Additional File 2. Target genes and primers for the housekeeping genes of *F. columnare.*** The loci used for the MLST/MLSA scheme are shown in bold font. Length refers to the length of the target sequence. .* Reference for 16S rDNA primers [52].

| Target gene | Gene product | Direction | primer sequence | Length |
| --- | --- | --- | --- | --- |
| *trpB* | **tryptophan synthase subunit B** | **Forward** | **5'- TGCCATACAGGTGCGCATAA -3'** | **730 bp** |
|  |  | **Reverse** | **5'- TGGCTCTCCCGCTTTTGAAT-3** |  |
| *gyrB* | **DNA gyrase B** | **Forward** | **5'-TACGCACGAAGGAGGAACAC-3'** | **704 bp** |
|  |  | **Reverse** | **5'-GGCTCCCATCAATATCGGCA-3'** |  |
| *dnaK* | **molecular chaperone DnaK** | **Forward** | **5'-GGCTACAGCTTCTGGACCAA-3'** | **727 bp** |
|  |  | **Reverse** | **5'-AGCGGCTTTATCTGCTTCCG-3'** |  |
| *tuf* | **Elongation factor Tu** | **Forward** | **5'-ACATGGTTACTGGTGCTGCT-3'** | **705 bp** |
|  |  | **Reverse** | **5'-TATGGAATGGCGTGTGACGA-3'** |  |
| *atpA* | **α-subunit of bacterial ATP synthase** | **Forward** | **5'-TGGACGTACCCCAGAGTTGA-3'** | **745 bp** |
|  |  | **Reverse** | **5'-GCGTAAAGCACCAGGGGTAA-3'** |  |
| *rpoD* | **RNA polymerase sigma factor** | **Forward** | **5'-AGCACAACGCATCAAGGCTGGT-3'** | **480 bp** |
|  |  | **Reverse** | **5'-TGGGGCATCCATAGATAAATGGCGT-3'** |  |
| *fumC* | fumarate hydratase | Forward | 5'-GTTCTAACCTGCCCGCTCAA-3' | 744 bp |
|  |  | Reverse | 5'-GCATTGGTTTGCCTGCATCT-3' |  |
| *murG* | N-acetylglucosaminyltransferases | Forward | 5'-GGGGAGGTACAGGAGGTCAT-3' | 767 bp |
|  |  | Reverse | 5'-AATGACTATATCGGCGGCGG-3' |  |
| *rplB* | 50S ribosomal protein L2 | Forward | 5'-CCTATTACCCCAGGTCAGCG-3' | 719 bp |
|  |  | Reverse | 5'-CTTGAACGTGGGTGACCTCC-3' |  |
| *recA* | recombinase A | Forward | 5'-GGCGGCTATCCTAAAGGTCG-3' | 746 bp |
|  |  | Reverse | 5'-CGGCCTTGACCGAGTTTTGT-3' |  |
| *fstQ* | cell division protein FtsQ | Forward | 5'-ACCCAAGTGGAAATTGTGGA-3' | 522bp |
|  |  | Reverse | 5'-TGTATCCTGGACTGCTTTTTGA-3' |  |
| *glyA* | serine hydroxy methyl transferase | Forward | 5'-AATGGAAGCGGCAGGTTCTT-3' | 705 bp |
|  |  | Reverse | 5'-GGGCCTCCTTGATTACCTGG-3' |  |
| *16S rDNA* | 16S ribosomal DNA | Forward (fD1)* | 5'-AGAGTTTGATCCTGGCTCAG-3' | 500 bp |
|  |  | Reverse (Prun 518) | 5'-ATTACCGCGGCTGCTGG-3' |  |
