# Supplementary material for: A multilocus sequence analysis scheme for characterization of Flavobacterium columnare isolates
Source: BMC Microbiol. 2015 Oct 30;15:243. doi: 10.1186/s12866-015-0576-4 (PMC4628280; doi:10.1186/s12866-015-0576-4)

Additional File 3. Neighbor Joining phylogenetic trees based on the individual sequences of six MLST loci (*trpB, rpoD, gyrB* , *dnaK, atpA*,and *tuf*). MEGA v5.2 was used to evaluate the models for nucleotide substitution for each protein-coding locus and to construct the phylogenetic trees for Finnish *F. columnare* strains. The *F. columnare* type strain NCIMB 2248^{T} isolated in the USA and two reference strains JIP39/87 and ATCC49512, both isolated in France, were also included in the phylogenetic analysis. The best model indicated by the lowest Bayesian Information Criterion (BIC) value was used to generate the Neighbor-Joining tree based on 1000 replicates. The T92 model was selected for *dnaK*, *tuf*, and *gyrB*, while T92+G was selected for both *trpB* and *rpoD* and JC model was selected for *atpA*. Strains corresponding to lineage I (cluster C and E) are coloured red and strains corresponding to lineage II (cluster G, A and H) are in blue. Other sequences assigned to reference strains are in black. Few strains representative of each genotype were used for tree construction (identical sequences removed for clarity of representation).

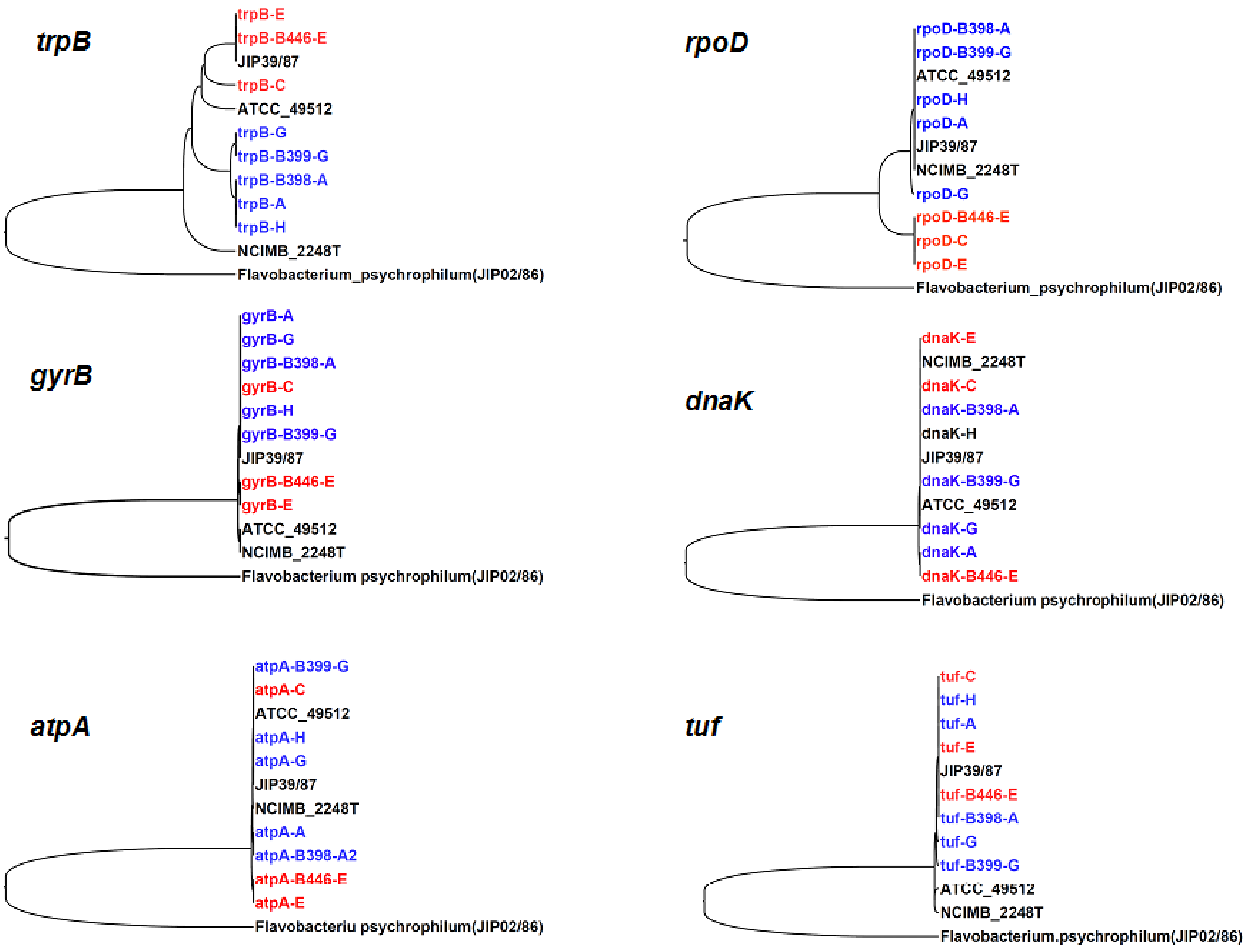

Supplement: Additional file 3 — Neighbor Joining phylogenetic trees based on the individual sequences of six MLST loci (trpB, rpoD, gyrB, dnaK, atpA,and tuf). MEGA v5.2 was used to evaluate the models for nucleotide substitution for each protein-coding locus and to construct the phylogenetic trees for Finnish F. columnare strains. The F. columnare type strain NCIMB 2248T isolated in the USA and two reference strains JIP39/87 and ATCC49512, both isolated in France, were also included in the phylogenetic analysis. The best model indicated by the lowest Bayesian Information Criterion (BIC) value was used to generate the Neighbor-Joining tree based on 1000 replicates. The T92 model was selected for dnaK, tuf, and gyrB, while T92 + G was selected for both trpB and rpoD and JC model was selected for atpA. Strains corresponding to lineage I (cluster C and E) are coloured red and strains corresponding to lineage II (cluster G, A and H) are in blue. Other sequences assigned to reference strains are in black. Few strains representative of each genotype were used for tree construction (identical sequences removed for clarity of representation). (PDF 120 kb) [file 12866_2015_576_MOESM3_ESM.pdf]
