# Supplementary material for: A multilocus sequence analysis scheme for characterization of Flavobacterium columnare isolates
Source: BMC Microbiol. 2015 Oct 30;15:243. doi: 10.1186/s12866-015-0576-4 (PMC4628280; doi:10.1186/s12866-015-0576-4)

Additional File 4. The fluorescence peak profiles for the ARISA genotypes analysed with ABI Prism 3130xl Genetic Analyser and the GeneMapper v.5.0 software (Applied Biosystems, Carlsbad, California, USA). For the 83 strains isolated from Finland, we also determined the ARISA (automated ribosomal intergenic spacer analysis) genotypes following the procedure described by Suomalainen et al.[8]. However, the previously published method was modified so that ABI Prism 3130xl Genetic Analyser is used instead of LI-COR 4200 automatic sequencer The analysis revealed that ARISA genotypes associate uniformly with the clusters from the MLSA scheme. Briefly, the PCR reaction mixture (total volume 10 ul) contained 1X DreamTaq Buffer (Thermo Scientific), 0.2 mM dNTPs (Thermo Scientific), 0.5 µM reverse primer (23Sr, 5'-GGGTTBCCCCATTCRG-3), 0.44 µM forward primer (rD1f, 5'-GGCTGGATCACCTCCTT-3'), 0.06 µM 6-carboxyfluorescein (6-FAM) labelled forward primer (rD1f), 1 U of DreamTaq DNA Polymerase (Thermo Scientific) and 1 µl of template DNA. The PCR reactions were carried out using Bio-Rad C1000 or S1000 thermal cyclers (Bio-Rad Laboratories, Hercules, CA, USA). The thermo-cycling conditions included initial denaturation at 95°C for 2 min followed by 30 cycles of amplification (94°C for 30 s, 52°C for 30 s, 72°C for 3 min) and a final extension at 72°C for 15 min. The PCR products were denatured with formamide and GeneScanTM 1200 LIZ Size Standard was added. Products were separated with an ABI Prism 3130xl Genetic Analyser, and visualized with GeneMapper v.5.0 software (all Applied Biosystems, Carlsbad, California, USA). Based on the fluorescence peak profiles and using the strains from Suomalainen et al. (8) as positive controls, the 87 *F. columnare* strains were designated into ARISA genotypes A to H [8].

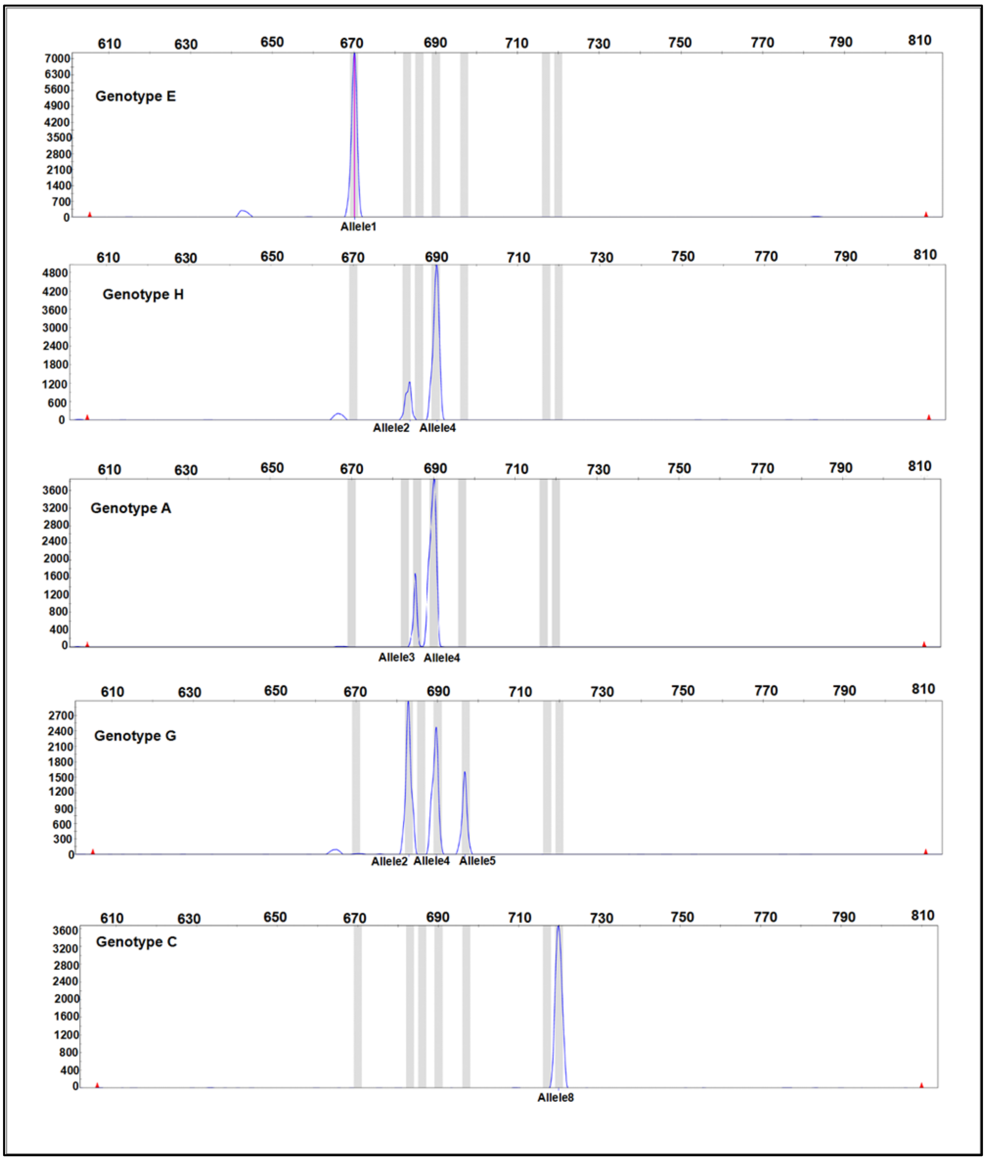

Supplement: Additional file 4 — The fluorescence peak profiles for the ARISA genotypes analysed with ABI Prism 3130xl Genetic Analyser and the GeneMapper v.5.0 software (Applied Biosystems, Carlsbad, California, USA). For the 83 strains isolated from Finland, we also determined the ARISA (automated ribosomal intergenic spacer analysis) genotypes following the procedure described by Suomalainen et al. [8]. However, the previously published method was modified so that ABI Prism 3130xl Genetic Analyser is used instead of LI-COR 4200 automatic sequencer The analysis revealed that ARISA genotypes associate uniformly with the clusters from the MLSA scheme. Briefly, the PCR reaction mixture (total volume 10 ul) contained 1X DreamTaq Buffer (Thermo Scientific), 0.2 mM dNTPs (Thermo Scientific), 0.5 μM reverse primer (23Sr, 5’-GGGTTBCCCCATTCRG-3), 0.44 μM forward primer (rD1f, 5’-GGCTGGATCACCTCCTT-3’), 0.06 μM 6-carboxyfluorescein (6-FAM) labelled forward primer (rD1f), 1 U of DreamTaq DNA Polymerase (Thermo Scientific) and 1 μl of template DNA. The PCR reactions were carried out using Bio-Rad C1000 or S1000 thermal cyclers (Bio-Rad Laboratories, Hercules, CA, USA). The thermo-cycling conditions included initial denaturation at 95 °C for 2 min followed by 30 cycles of amplification (94 °C for 30 s, 52 °C for 30 s, 72 °C for 3 min) and a final extension at 72 °C for 15 min. The PCR products were denatured with formamide and GeneScanTM 1200 LIZ Size Standard was added. Products were separated with an ABI Prism 3130xl Genetic Analyser, and visualized with GeneMapper v.5.0 software (all Applied Biosystems, Carlsbad, California, USA). Based on the fluorescence peak profiles and using the strains from Suomalainen et al. [8] as positive controls, the 87 F. columnare strains were designated into ARISA genotypes A to H [8]. (PDF 222 kb) [file 12866_2015_576_MOESM4_ESM.pdf]
