# Supplementary material for: A multilocus sequence analysis scheme for characterization of Flavobacterium columnare isolates
Source: BMC Microbiol. 2015 Oct 30;15:243. doi: 10.1186/s12866-015-0576-4 (PMC4628280; doi:10.1186/s12866-015-0576-4)

Additional File 5. Phylogenetic tree based on the 16s rDNA sequence data obtained from the representatives of Finnish *F .columnare* genotypes (A-H) studied in this study and other *F .columnare* sequences obtained from the GenBank. The tree was constructed by a UPGMA clustering method with a resampling of 1,000 bootstrap replicates and the Jukes Cantor model. Two strains representative of each ARISA genotype/MLSA cluster studied in this study were used for tree construction (identical sequences removed for clarity of representation).

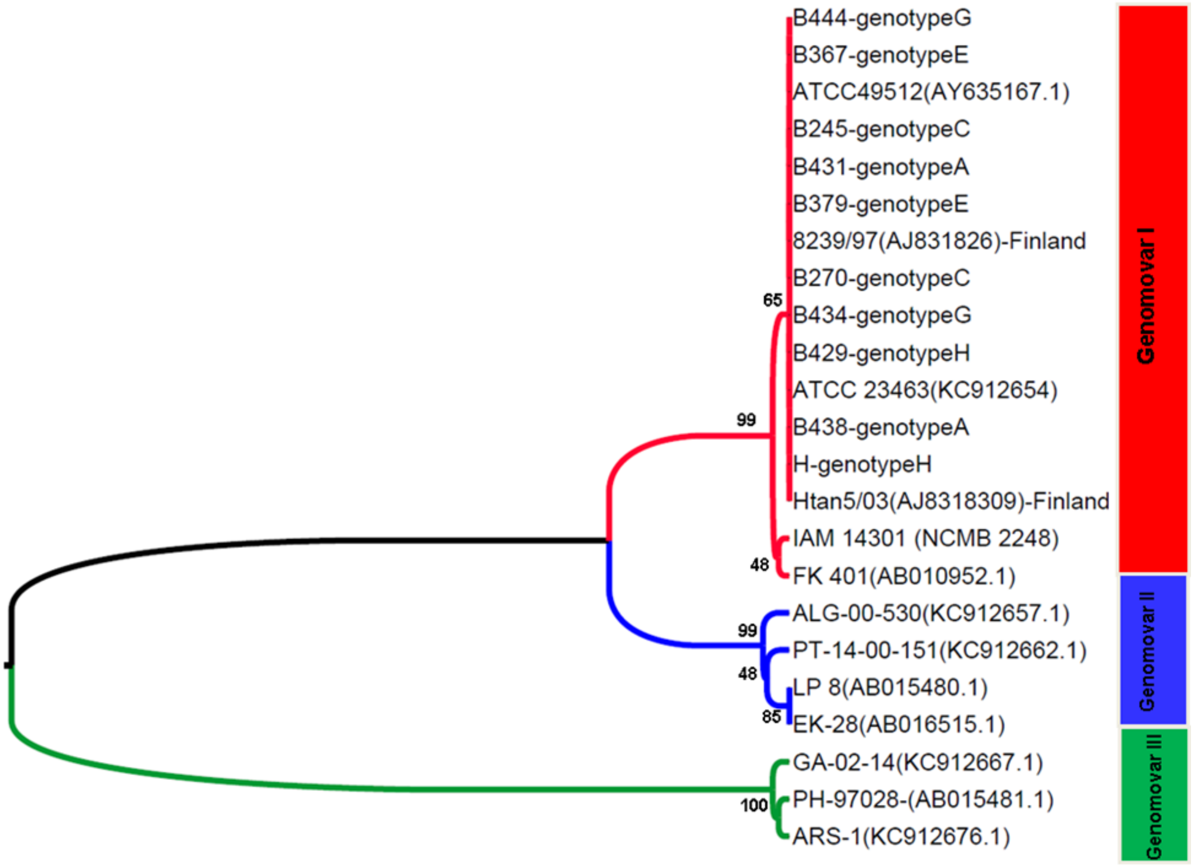

Supplement: Additional file 5 — Phylogenetic tree based on the 16 s rDNA sequence data obtained from the representatives of Finnish F .columnare genotypes (A-H) studied in this study and other F .columnare sequences obtained from the GenBank. The tree was constructed by a UPGMA clustering method with a resampling of 1,000 bootstrap replicates and the Jukes Cantor model. Two strains representative of each ARISA genotype/MLSA cluster studied in this study were used for tree construction (identical sequences removed for clarity of representation). (PDF 191 kb) [file 12866_2015_576_MOESM5_ESM.pdf]
