# Supplementary material for: A multilocus sequence analysis scheme for characterization of Flavobacterium columnare isolates
Source: BMC Microbiol. 2015 Oct 30;15:243. doi: 10.1186/s12866-015-0576-4 (PMC4628280; doi:10.1186/s12866-015-0576-4)

Additional File 6. The concatenated tree based on six housekeeping MLSA genes (including *trpB;* right) and five MLSA gene sequences (*rpoD*, *dnaK*, *tuf*, *gyrB*, *atpA*; left). The arrow shows disagreement concerning the position of strain B399-G between the two trees.

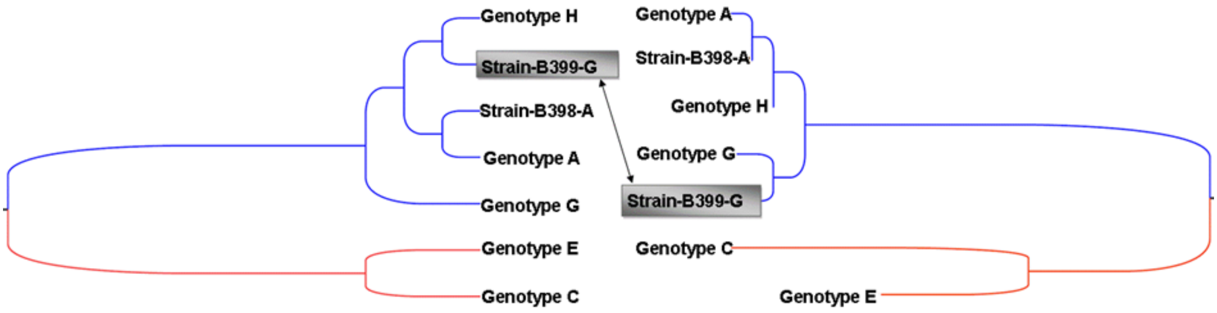

Supplement: Additional file 6 — The concatenated tree based on six housekeeping MLSA genes (including trpB; right) and five MLSA gene sequences (rpoD, dnaK, tuf, gyrB, atpA; left). The arrow shows disagreement concerning the position of strain G2 (B399) between the two trees. (PDF 77 kb) [file 12866_2015_576_MOESM6_ESM.pdf]
